# Frailty index predicts hearing loss in middle-aged and older adults: A triangulated analysis of global, cohort, and genetic data

**DOI:** 10.1016/j.bjorl.2026.101919

**Published:** 2026-10-17

**Authors:** Liang Ren, Xiangling Jiang, Maohua Wang, Xu Zhang

**Affiliations:** a Ankang Gaoxin Hospital, Department of Otorhinolaryngology Head and Neck Surgery, Ankang, Shanxi Province, China; b The First People’s Hospital of Foshan, Hearing and Balance Medical Engineering Technology Center of Guangdong, Department of Otolaryngology, Head and Neck Surgery, Foshan, China; c Nanchang University, Jiangxi Medical College, The Second Affiliated Hospital, Department of Otorhinolaryngology Head and Neck Surgery, Nanchang, Jiangxi Province, China

**Keywords:** Frailty, Frailty index, Hearing loss, CHARLS, Risk prediction, Mendelian randomization

## Abstract

**Objective:**

To examine the association between frailty (Frailty Index, FI) and incident hearing loss using triangulation across GBD 2021, CHARLS, and Mendelian Randomization (MR).

**Methods:**

Global trends were analyzed with GBD 2021. In CHARLS (2011–2020), FI was calculated for adults ≥ 45-years. Associations with incident hearing loss were assessed using logistic regression, restricted cubic splines, and ROC curves. Two-sample MR employed inverse-variance weighted, weighted median, and MR-Egger methods.

**Results:**

GBD revealed increasing hearing loss burden globally, especially among older adults in low- and middle-income countries. In CHARLS, higher FI independently predicted incident hearing loss: moderate frailty increased odds by 66% (OR = 1.66, 95% CI: 1.29–2.12) and severe frailty by 141% (OR = 2.41, 95% CI: 1.47–3.95). Each unit FI increase conferred a 9% higher risk (OR = 1.09, 95% CI: 1.04–1.14). RCS identified a nonlinear threshold (FI ≈ 0.29). Adding FI to conventional risk factors improved prediction (AUC: 0.586 to 0.685). MR supported a causal effect of genetically predicted frailty on hearing loss with no evidence of pleiotropy.

**Conclusion:**

The Frailty Index is a robust, independent, and nonlinear predictor of incident hearing loss. Integrating frailty assessment enhances early identification and supports targeted prevention.

**Level of evidence:**

II.

## Highlights

• Triangulated evidence of frailty & hearing loss.
• Strong, non-linear predictor of hearing loss.
• Frailty index enhances hearing risk prediction.
• Genetic evidence supports causal role of frailty.
• Clinical relevance for aging populations.

## Introduction

Hearing loss is one of the most common and disabling chronic conditions worldwide, with profound consequences for communication, cognitive decline, social isolation, depression, and decreased quality of life, especially among older adults.^1^^,^^2^ According to the Global Burden of Disease (GBD) 2021 study, the prevalence and Years Lived with Disability (YLDs) attributed to hearing loss have been steadily increasing over the past three decades, making it a substantial global public health challenge. In China ‒ a country with the world’s largest aging population ‒ the burden of hearing loss is particularly acute and projected to rise further, stressing the urgent need for preventive strategies tailored to at-risk {Citation}populations. Frailty is a multidimensional syndrome characterized by the accumulation of deficits in physiological, psychological, and social domains, leading to increased vulnerability to various adverse health outcomes, including disability, hospitalization, and mortality.^3^ The Frailty Index (FI), based on the proportion of health deficits present in an individual, has emerged as a reliable and quantifiable measure of frailty status.^4^ In recent years, evidence has accumulated that frailty is intricately linked not only to traditional geriatric syndromes but also to sensory impairments, such as vision and hearing loss.^5^ However, the exact nature and strength of the relationship between frailty and incident hearing loss ‒ especially in large, representative populations-remain underexplored. Given that both frailty and hearing loss share common pathogenic pathways, including chronic inflammation, vascular dysfunction, metabolic dysregulation, and neurodegeneration, understanding their interplay is crucial for early identification and effective prevention of hearing decline in older adults.^3^ The China Health and Retirement Longitudinal Study (CHARLS), with its nationally representative cohort and detailed health assessments, offers a unique opportunity to examine these associations in Chinese populations. Likewise, the GBD database provides valuable context on epidemiologic trends and the global burden across multiple regions and time periods. Therefore, this study aimed to systematically evaluate the association between frailty-as measured by the Frailty Index-and the risk of incident hearing loss in middle-aged and older adults, integrating results from both the CHARLS cohort and the GBD database. The findings are expected to inform targeted prevention strategies and resource allocation in the context of healthy aging.

## Methods

### Data source and study population

This study integrates data from three complementary sources: the 2021 Global Burden of Disease (GBD) database, the China Health and Retirement Longitudinal Study (CHARLS), and Genome-Wide Association Study (GWAS) summary statistics for Mendelian Randomization (MR) analysis. GBD 2021 data were obtained from the open-access Global Health Data Exchange (GHDx) Results Tool (http://ghdx.healthdata.org/), providing standardized estimates of hearing loss incidence, prevalence, and Years Lived with Disability (YLDs) across 204 countries and territories from 1990 to 2021. Detailed GBD methodology is described elsewhere. As aggregated public data, the GBD database does not require ethical approval. CHARLS is a nationally representative longitudinal cohort initiated in 2011, enrolling Chinese adults aged ≥ 45 years through multistage, stratified, probability-proportional-to-size sampling across 28 provinces. Biennial follow-up assessments were conducted through 2020, completing five survey rounds. Participants were included if they: 1) Were aged ≥ 45-years at baseline; 2) Completed baseline and ≥ 1 follow-up assessment; 3) Had no hearing loss at baseline; and 4) Had complete data for Frailty Index calculation and all covariates. Participants with pre-existing hearing loss or missing data were excluded. Sample selection is detailed in Fig. 1. For MR analysis, GWAS summary statistics were obtained from publicly available databases to examine causal relationships between frailty and hearing loss. Genetic instruments for frailty (exposure) were derived from large-scale GWAS meta-analyses, while outcome data for hearing loss-related phenotypes were sourced from the UK Biobank and European Bioinformatics Institute (EBI) GWAS Catalog, including multiple hearing impairment definitions and audiometric measures. All GWAS datasets utilized European ancestry populations to minimize population stratification bias. Instrumental variables were selected based on genome-wide significance (p < 5 × 10^−8^), independence (linkage disequilibrium *r*² < 0.001 within 10,000 kb), and availability in outcome datasets. MR analysis employed publicly available summary statistics and did not require additional ethical approval.

**Fig. 1:**
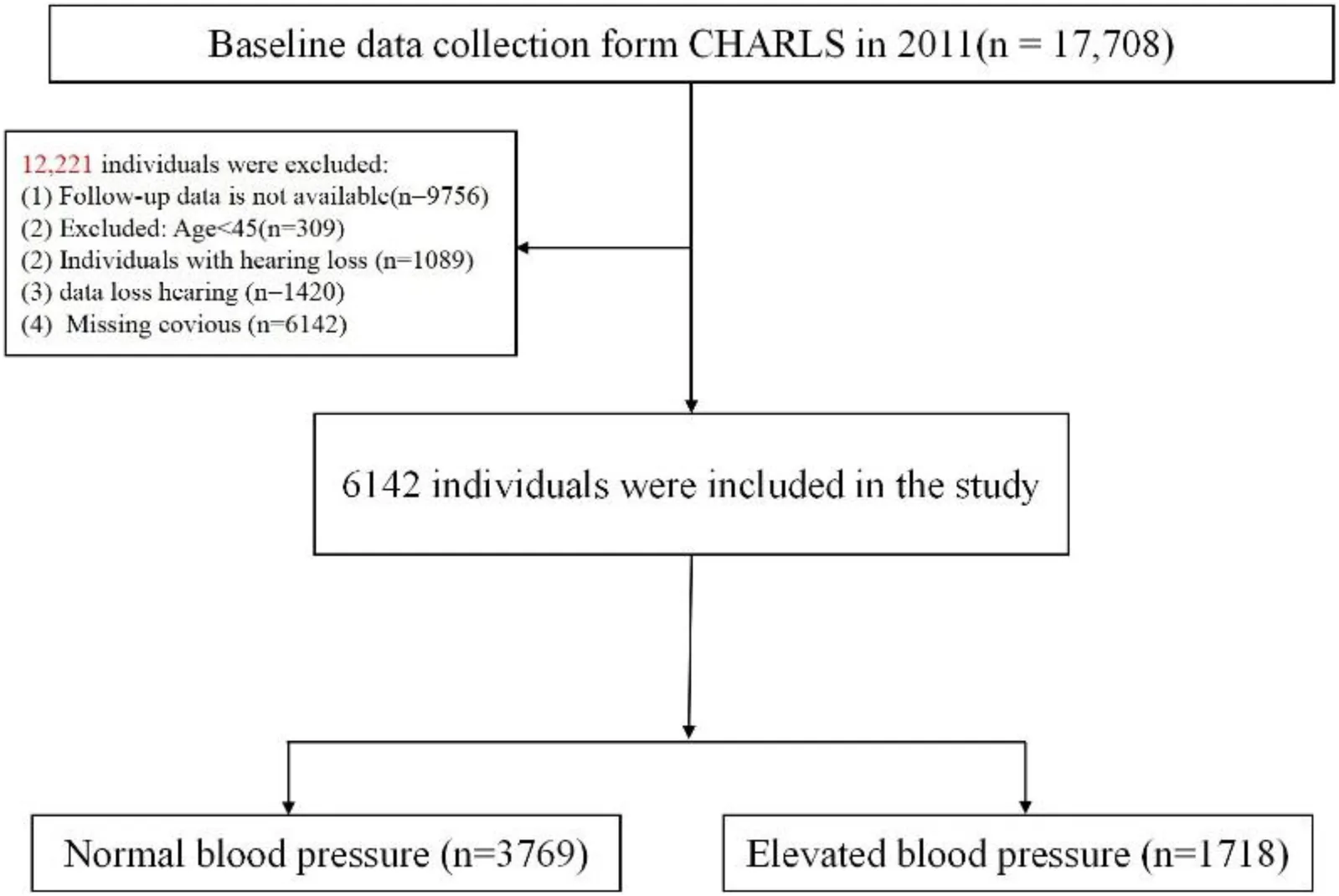
Flowchart of participant selection from the CHARLS cohort.

### Data collection and calculation of frailty index

All baseline and follow-up data were collected by trained staff using standardized protocols. Key variables included: Demographics: Age, sex, residence (urban/rural), education (illiterate, junior high school or below, high school or above), marital status. Lifestyle factors: Smoking and alcohol consumption status. Anthropometric and laboratory measures: BMI, waist circumference, blood pressure, total cholesterol, triglycerides, and low-density lipoprotein, measured per national guidelines. Comorbidities: Self-reported or physician-confirmed hypertension, diabetes, cardiovascular disease, stroke, and dyslipidemia

Frailty was operationalized using the validated deficit accumulation approach. For each participant, 30–40 age-related health deficits spanning functional status, comorbidities, psychological symptoms, cognition, and self-reported health were identified following established recommendations.^3^^,^^6^ Each deficit was scored as 0 (absent), 1 (present), or intermediate values (0.5) for ordinal responses. The Frailty Index (FI) was calculated as: ${\text{F}\text{I}}_{j}=\frac{1}{n}\sum_{i=1}^{n}d_{ij},0\leq d_{ij}\leq 1$.

The FI ranges from 0 (no frailty) to 1 (maximal frailty), with population values typically between 0‒0.5. Deficit selection criteria required health-related conditions with age-increasing prevalence, broad system coverage, and < 5% missing values. The FI was analyzed both continuously and categorically by quartiles (Q1‒Q4) for risk modeling.

### Outcome definition

Incident hearing loss was defined as new-onset hearing impairment in participants with normal hearing at baseline, identified through either self-reported physician diagnosis or objective hearing assessment during follow-up. Participants with baseline hearing loss were excluded from longitudinal analyses.

### Logistic regression modeling

Multiple logistic regression models were constructed to assess associations between FI and incident hearing loss, with FI analyzed both as a continuous variable (per 0.01-unit increase) and categorically by quartiles. Four progressive models were developed with increasing covariate adjustment: a crude model included FI alone, while Model 1 adjusted for demographic factors including age, sex, residence, and education. Model 2 further incorporated lifestyle and metabolic factors, adding marital status, smoking, alcohol consumption, BMI, waist circumference, total cholesterol, LDL cholesterol, and triglycerides to the previous model. The fully adjusted Model 3 additionally controlled for comorbidities including stroke, diabetes, cardiovascular disease, dyslipidemia, and age group stratification. Odds ratios with 95% Confidence Intervals were calculated for all models, and p-values for trend were computed to assess dose-response relationships across FI quartiles.

### Nonlinear association analysis

Restricted Cubic Spline (RCS) regression explored potential nonlinear relationships between FI and hearing loss risk. Model selection utilized Bayesian Information Criterion, with likelihood ratio tests assessing nonlinearity. RCS plots visualized risk functions and inflection points, adjusting for covariates in the fully adjusted model.

### Predictive performance assessment

Receiver Operating Characteristic (ROC) curves evaluated discriminative performance of FI alone and combined with other risk factors. Area Under the Curve (AUC), sensitivity, specificity, and optimal cut-off values were calculated for all models. Predictive improvements with covariate adjustment were assessed statistically and graphically.

### Mendelian randomization analysis

Two-sample Mendelian randomization was conducted to examine causal relationships between genetically predicted frailty and hearing loss phenotypes. Genetic instruments for frailty were selected from large-scale GWAS meta-analyses based on genome-wide significance (p < 5 × 10^−8^), independence (linkage disequilibrium *r*² < 0.001 within 10,000 kb window), and relevance to the exposure.^7^, ^8^, ^9^ Instrumental variable strength was assessed using F-statistics, with F > 10 indicating adequate instrument strength to minimize weak instrument bias. The primary MR analysis employed the Inverse-Variance Weighted (IVW) method, assuming all genetic variants are valid instruments. Complementary methods included MR-Egger regression to detect and adjust for directional pleiotropy, weighted median estimation robust to up to 50% invalid instruments, and MR-PRESSO to identify and remove outlier variants. Heterogeneity was evaluated using Cochran's *Q* statistic and *I*^2^ index, while horizontal pleiotropy was assessed through MR-Egger intercept tests. Sensitivity analyses included leave-one-out analysis to identify influential variants and reverse MR to explore bidirectional causality.^3^

### Statistical analysis

All analyses were conducted with R (version 4.2.2). Normality of continuous variables was assessed via the Shapiro-Wilk test. If variables departed from normality, they were summarized as median (Interquartile Range, IQR); categorical variables were presented as n (%). Between-group differences were tested using the Mann-Whitney *U*-test for continuous variables and Chi-Square or Fisher’s exact test for categorical variables.

## Results

### Global and national trends of hearing loss (GBD 2021 data)

Global hearing loss burden increased substantially from 1990 to 2021, with age-standardized prevalence rising from 11.3% to 15.9% and associated Years Lived with Disability (YLDs) increasing from 15.8 to 23.3 million-a 47.2% relative increase. This burden disproportionately affected low- and middle-income countries and individuals aged ≥60-years. China exhibited a similar trajectory, with age-standardized prevalence increasing from 10.1% to 14.7% during the same period. The Estimated Annual Percentage Change (EAPC) in prevalence was most pronounced among Chinese men and adults aged ≥ 60-years, with China's overall rate of increase (EAPC: 1.32, 95% CI: 1.12–1.51) slightly exceeding the global average. Subnational analysis revealed heterogeneous patterns within China, with central and western provinces demonstrating higher hearing loss burden-regions that coincidentally exhibit elevated frailty prevalence and accelerated population aging (Fig. S1, Table S1‒2).

### Baseline characteristics of study population

Of the 6142 CHARLS participants meeting inclusion criteria, the mean baseline age was 57.5 ± 8.3 years, with 55.8% women and 65.0% residing in rural areas. During a median follow-up period, 412 (6.7%) participants developed incident hearing loss. Compared to those who remained hearing-intact (n = 5730), participants who developed hearing loss were significantly older (61.2 ± 8.9 vs. 57.2 ± 8.2 years, p < 0.0001) with a higher proportion in older age groups (54.2% aged ≥ 60-years vs. 39.2%, p < 0.0001). Those experiencing hearing loss demonstrated significantly higher baseline frailty burden, with mean deficit count of 29.4 ± 2.1 vs. 28.7 ± 2.5 (p < 0.0001) and greater prevalence of pre-frailty and frailty states (46.6% vs. 30.2%, p < 0.0001). The incident hearing loss group was characterized by lower educational attainment (37.1% illiterate vs. 25.8%, p < 0.001), higher rural residence (72.3% vs. 64.5%, p < 0.01), and increased prevalence of comorbidities including cardiovascular disease (20.4% vs. 10.1%, p < 0.0001), lung diseases (12.4% vs. 8.7%, p = 0.02), and liver disease (5.8% vs. 3.0%, p < 0.01). Notably, participants who developed hearing loss had lower BMI (23.3 ± 3.9 vs. 23.8 ± 3.9 kg/m^2^, p = 0.01), while other metabolic parameters showed no significant differences. Complete baseline characteristics are presented in Table 1.

**Table 1:** Baseline characteristics of study population.

Variable	Total (n = 6142)	No (n = 5730)	Yes (n = 412)	Statistic	p-value
Sex				0.24	0.62
Female	3424 (55.78)	3200 (55.88)	224 (54.50)		
Male	2714 (44.22)	2527 (44.12)	187 (45.50)		
Age	57.50 ± 8.26	57.23 ± 8.15	61.22 ± 8.86	−8.86	<0.0001
Age group				80.74	<0.0001
45‒49	1034 (19.11)	999 (19.80)	35 (9.56)		
50‒59	2188 (40.43)	2069 (41.00)	119 (32.51)		
60–69	1648 (30.45)	1499 (29.71)	149 (40.71)		
70‒79	496 (9.16)	446 (8.84)	50 (13.66)		
80+	46 (0.85)	33 (0.65)	13 (3.55)		
Marital status				0.68	0.41
Married	5308 (86.42)	4958 (86.53)	350 (84.95)		
Non-married	834 (13.58)	772 (13.47)	62 (15.05)		
Education				15.14	<0.001
High school or above	674 (10.97)	650 (11.34)	24 (5.83)		
Illiterate	1607 (26.16)	1478 (25.79)	129 (31.31)		
Junior high school or below	3861 (62.86)	3602 (62.86)	259 (62.86)		
Residence place				10.01	<0.01
Rural	3994 (65.03)	3696 (64.50)	298 (72.33)		
Urban	2148 (34.97)	2034 (35.50)	114 (27.67)		
Fi				58.83	<0.0001
Frailty	168 (2.74)	141 (2.46)	27 (6.55)		
Pre-farilty	1756 (28.59)	1591 (27.77)	165 (40.05)		
Robust	4218 (68.67)	3998 (69.77)	220 (53.40)		
Count	28.74 ± 2.44	28.69 ± 2.46	29.38 ± 2.07	−6.38	<0.0001
BMI	23.76 ± 3.85	23.79 ± 3.85	23.29 ± 3.90	2.51	0.01
Waist	84.53 ± 12.45	84.61 ± 12.39	83.45 ± 13.25	1.72	0.09
Glucose	109.12 ± 33.51	109.12 ± 33.88	109.09 ± 27.92	0.02	0.98
Uric	4.41 ± 1.23	4.41 ± 1.22	4.41 ± 1.26	0.01	1.00
TC	5.02 ± 0.98	5.02 ± 0.98	5.06 ± 1.02	−0.89	0.38
LDL	3.03 ± 0.90	3.03 ± 0.90	3.00 ± 0.90	0.55	0.59
TG	1.50 ± 1.09	1.50 ± 1.08	1.50 ± 1.14	−0.08	0.94
Smoke				1.45	0.23
No	4342 (70.69)	4062 (70.89)	280 (67.96)		
Yes	1800 (29.31)	1668 (29.11)	132 (32.04)		
Drinking				1.32	0.25
No	4116 (67.01)	3851 (67.21)	265 (64.32)		
Yes	2026 (32.99)	1879 (32.79)	147 (35.68)		
Hypertension				0.50	0.48
No	4716 (76.78)	4406 (76.89)	310 (75.24)		
Yes	1426 (23.22)	1324 (23.11)	102 (24.76)		
Lung diseases				5.91	0.02
No	5592 (91.05)	5231 (91.29)	361 (87.62)		
Yes	550 (8.95)	499 (8.71)	51 (12.38)		
Liver				9.52	<0.01
No	5949 (96.86)	5561 (97.05)	388 (94.17)		
Yes	193 (3.14)	169 (2.95)	24 (5.83)		
Stroke				2.84	0.09
No	6035 (98.26)	5635 (98.34)	400 (97.09)		
Yes	107 (1.74)	95 (1.66)	12 (2.91)		
DM				1.01	0.32
No	5339 (86.93)	4988 (87.05)	351 (85.19)		
Yes	803 (13.07)	742 (12.95)	61 (14.81)		
CVD				40.96	<0.0001
No	5478 (89.19)	5150 (89.88)	328 (79.61)		
Yes	664 (10.81)	580 (10.12)	84 (20.39)		
Dyslipidemia				0.01	0.92
No	3645 (59.35)	3402 (59.37)	243 (58.98)		
Yes	2497 (40.65)	2328 (40.63)	169 (41.02)		
BMI group				5.23	0.07
Normal	4119 (67.06)	3823 (66.72)	296 (71.84)		
Obesity	309 (5.03)	288 (5.03)	21 (5.10)		
Overweight	1714 (27.91)	1619 (28.25)	95 (23.06)		

### Incidence and risk of hearing loss by frailty index (CHARLS data)

Over the follow-up period, a clear dose-response relationship emerged between baseline frailty status and incident hearing loss risk. Using the established frailty classification system, participants classified as pre-frail (Category 1) demonstrated significantly higher risk compared to robust individuals (Category 0), while those classified as frail (Category 2) showed the highest risk across all analytical models. In the crude model, both pre-frail and frail participants showed substantial increases in hearing loss risk compared to robust individuals, with odds ratios of 1.88 (95% CI: 1.53–2.33, p < 0.0001) and 3.48 (95% CI: 2.26–5.37, p < 0.0001), respectively. After adjusting for demographic factors (Model 1), these associations remained robust: pre-frail OR = 1.70 (95% CI: 1.37–2.11, p < 0.0001) and frail OR = 2.67 (95% CI: 1.71–4.17, p < 0.0001). Further adjustment for lifestyle factors, anthropometric measures, and metabolic parameters (Model 2) yielded similar estimates, with pre-frail OR = 1.75 (95% CI: 1.41–2.18, p < 0.0001) and frail OR = 2.88 (95% CI: 1.83–4.53, p < 0.0001). In the fully adjusted model (Model 3) incorporating all baseline comorbidities, the association remained significant with adjusted ORs of 1.66 (95% CI: 1.29–2.12, p < 0.0001) for pre-frail and 2.41 (95% CI: 1.47–3.95, p < 0.001) for frail participants, demonstrating a consistent dose-response pattern (p for trend < 0.0001 across all models). When analyzed as a continuous variable, each unit increase in the frailty deficit count conferred a significant increase in hearing loss risk. In the crude model, the OR was 1.13 (95% CI: 1.08–1.19, p < 0.0001), which remained consistent after full adjustment at 1.09 (95% CI: 1.04–1.14, p < 0.001). Using inter-quartile range increments, the association was even more pronounced, with fully adjusted OR = 1.52 (95% CI: 1.19–1.93, p < 0.001), indicating that clinically meaningful increases in frailty burden substantially elevate hearing loss risk. Quartile-based analysis revealed an interesting pattern where the middle quartiles showed the strongest associations with hearing loss. Compared to Q1 (lowest frailty), participants in Q2 demonstrated consistently elevated risk across all models, with the fully adjusted OR = 2.10 (95% CI: 1.58–2.79, p < 0.0001). Q3 participants also showed significant associations, with fully adjusted OR = 1.51 (95% CI: 1.13–2.02, p = 0.01). Notably, Q4 (highest frailty) showed non-significant associations in adjusted models (OR = 0.99, 95% CI: 0.44–2.23, p = 0.99), suggesting a potential threshold effect or competing risks in the most frail individuals. The trend analysis using median quartile values-maintained significance across all models (p < 0.001 in Model 3), while the categorical trend showed some attenuation in the fully adjusted model (p = 0.08), likely reflecting the complex relationship at the highest frailty levels. These findings demonstrate that frailty represents a robust and independent predictor of incident hearing loss, with the relationship being most pronounced in the pre-frail to moderately frail range. The consistent associations across multiple analytical approaches and adjustment strategies underscore frailty as a clinically relevant risk factor that could inform hearing loss prevention strategies, particularly in the pre-frail population where intervention potential may be greatest (Fig. 2, Table 2).

**Fig. 2:**
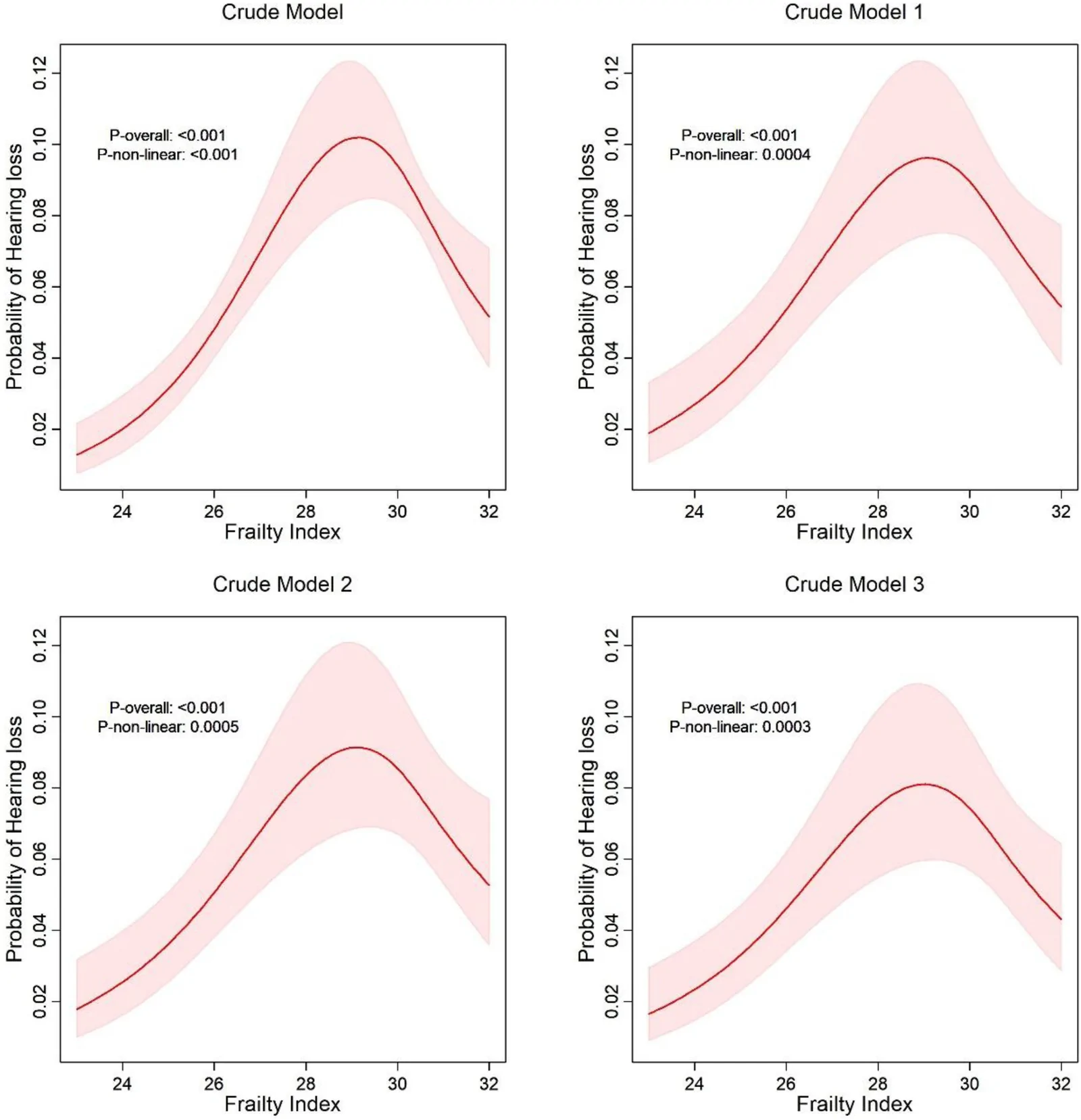
Association between frailty index and incident hearing loss.

**Table 2:** Association between hearing status and frailty: crude and adjusted models.

Character	Crude model		Model 1		Model 2		Model 3	
	95%CI	p	95%CI	p	95%CI	p	95%CI	p
**Hear ∼ Frilty**								
Robust	Ref		Ref		Ref		Ref	
Pre-frailty	1.88 (1.53, 2.33)	<0.0001	1.7 (1.37, 2.11)	<0.0001	1.75 (1.41, 2.18)	<0.0001	1.66 (1.29, 2.12)	<0.0001
Frailty	3.48 (2.26, 5.37)	<0.0001	2.67 (1.71, 4.17)	<0.0001	2.88 (1.83, 4.53)	<0.0001	2.41(1.47, 3.95)	<0.001
p for trend (character2integer)		<0.0001		<0.0001		<0.0001		<0.0001
**Hear ∼ Frilty Index**	1.13 (1.08, 1.19)	<0.0001	1.1 (1.05, 1.15)	<0.0001	1.1 (1.05, 1.16)	<0.0001	1.09 (1.04, 1.14)	<0.001
**Hear ∼ Frilty Index_IQR**	1.87 (1.49, 2.34)	<0.0001	1.6 (1.26, 2.03)	<0.0001	1.63 (1.29, 2.07)	<0.0001	1.52 (1.19, 1.93)	<0.001
**Hear ∼ Frilty Index Q**								
Q1	ref		ref		ref		ref	
Q2	2.72 (2.09, 3.53)	<0.0001	2.22 (1.68, 2.94)	<0.0001	2.24 (1.69, 2.96)	<0.0001	2.1 (1.58, 2.79)	<0.0001
Q3	1.83 (1.38, 2.41)	<0.0001	1.6 (1.21, 2.13)	0.001	1.63 (1.22, 2.17)	<0.001	1.51 (1.13, 2.02)	0.01
Q4	1.31 (0.60, 2.90)	0.50	1.14 (0.51, 2.53)	0.75	1.2 (0.54, 2.67)	0.66	0.99 (0.44, 2.23)	0.99
p for trend (character2integer)		<0.001		0.02		0.01		0.08
p for trend (Median value)		<0.0001		<0.0001		<0.0001		<0.001

Crudel model: Frilty; Model 1: Frilty, sex, age, residence_place, marital_status, education; Model 2: Frilty, sex, age, residence_place, marital_status, education, BMI, waist, glucose, uric, TC, LDL, TG, smoke, drinking, BMI_group; Model 3: Frilty, sex, age, residence_place, marital_status, education, BMI, waist, glucose, uric, TC, LDL, TG, smoke, drinking, BMI_group, hypertension, lung_diseases, liver, stroke, DM, CVD, dyslipidemia.

### Nonlinear association and threshold effects

Restricted cubic spline modeling revealed a significant nonlinear association between FI and hearing loss risk across all analytical models. The overall association remained highly significant (p-overall < 0.001) in all four models, while the nonlinear component showed varying degrees of significance: crude model (p-non-linear < 0.001), Model 1 (p-non-linear = 0.0004), Model 2 (p-non-linear = 0.0005), and the fully adjusted Model 3 (p-non-linear = 0.0003). The spline curves consistently demonstrated an inverted *U*-shaped relationship, with hearing loss probability initially increasing with higher frailty index values, reaching a peak FI = 28–29, and then plateauing or slightly declining at the highest frailty levels. This pattern suggests a threshold effect where the marginal impact of additional frailty burden diminishes beyond a critical point. To formally test for threshold effects, we performed piecewise linear regression analysis, which identified an optimal inflection point at FI = 29. The two-segment model demonstrated significantly better fit compared to standard logistic regression.

The piecewise analysis revealed distinctly different associations in the two segments: below the threshold (FI < 29), the association between frailty and hearing loss was not statistically significant (OR = 0.913, 95% CI: 0.602–1.385, p = 0.669), while above the threshold (FI ≥ 29), higher frailty was paradoxically associated with lower hearing loss risk (OR = 0.703, 95% CI: 0.574–0.862, p < 0.001). This unexpected finding in the higher frailty range may reflect competing risks, survival bias, or measurement challenges in the most severely frail individuals. The identification of FI = 29 as a critical threshold has important clinical implications. This cut point, corresponding to approximately 29 health deficits out of the assessed domains, may represent a transition point where different biological mechanisms predominate. Below this threshold, the standard linear relationship between frailty and hearing loss risk applies, consistent with our continuous variable analysis (OR = 1.087, 95% CI: 1.035–1.141, p < 0.001). However, beyond this point, the relationship becomes more complex, possibly due to competing health priorities, different care patterns, or survival effects in the most frail individuals. These findings suggest that hearing loss prevention strategies may be most effective when targeting individuals in the pre-threshold range (FI < 29), where the relationship between frailty and hearing risk remains predictable and potentially modifiable. The threshold effect also highlights the importance of comprehensive geriatric assessment in identifying individuals at different stages of the frailty trajectory (Fig. S2).

### Subgroup and interaction analyses

Stratified analyses demonstrated remarkable consistency in the frailty-hearing loss association across diverse subgroups. Using frailty index as a continuous variable, the association remained statistically significant in virtually all examined strata, with adjusted ORs consistently ranging from 1.103 to 1.171. The positive association was robust across demographic factors: women (OR = 1.154, 95% CI: 1.078–1.240) and men (OR = 1.129, 95% CI: 1.063–1.201) showed similar associations, as did urban (OR = 1.122, 95% CI: 1.037–1.219) and rural residents (OR = 1.135, 95% CI: 1.076–1.201). Educational level showed consistent patterns, with strongest associations in those with junior high school education or below (OR = 1.133, 95% CI: 1.073–1.200, p < 0.0001) and significant associations even in illiterate participants (OR = 1.103, 95% CI: 1.011–1.212, p = 0.033). Lifestyle factors including smoking and drinking status did not modify the relationship, with both smokers and non-smokers, as well as drinkers and non-drinkers, showing significant associations (all p < 0.001). The frailty-hearing loss relationship remained robust across various health conditions, with significant associations observed in participants both with and without major comorbidities including hypertension, diabetes, and cardiovascular disease. Age-stratified analysis revealed the strongest association in the 60–69 years group (OR = 1.129, 95% CI: 1.043–1.230, p = 0.004), while other age groups showed variable patterns. Among BMI categories, normal-weight participants demonstrated the most pronounced association (OR = 1.157, 95% CI: 1.098–1.221, p < 0.0001). The consistency of associations across demographic, lifestyle, and clinical subgroups supports the clinical utility of frailty assessment as a broadly applicable screening tool for hearing loss risk, with predictive value that transcends traditional risk stratification approaches (Fig. S3).

### Predictive performance of frailty index and multivariable models

Receiver Operating Characteristic (ROC) curve analyses revealed that FI demonstrated superior discriminatory ability compared to individual traditional risk factors. FI alone achieved an AUC of 0.586 (95% CI: 0.561–0.612) with moderate sensitivity (47%) and good specificity (70%) at the optimal cut-off of 0.073. In contrast, conventional metabolic markers showed poor discriminatory performance: Triglycerides (TG) yielded an AUC of only 0.516 (95% CI: 0.487–0.546) with 56% sensitivity and 50% specificity, while Diabetes Mellitus (DM) performed even worse with AUC of 0.509 (95% CI: 0.492–0.527), demonstrating high specificity (87%) but very low sensitivity (15%). Sequential model building demonstrated substantial improvements in predictive accuracy with the addition of covariates to the baseline frailty model. The frailty-only model (Model 0) served as the foundation with AUC = 0.586 (95% CI: 0.561–0.612), achieving 46.6% sensitivity and 69.8% specificity. Addition of demographic variables (Model 1) yielded a notable improvement to AUC = 0.662 (95% CI: 0.635–0.689), with substantially increased sensitivity (71.5%) but reduced specificity (52.0%), suggesting enhanced case detection at the cost of more false positives. Further incorporation of lifestyle factors and laboratory markers (Model 2) continued the upward trend with AUC = 0.672 (95% CI: 0.645–0.699), achieving a more balanced performance profile (sensitivity 55.0%, specificity 70.3%). The fully adjusted model (Model 3), incorporating disease history and comorbidities, achieved the best overall discriminatory performance with AUC = 0.685 (95% CI: 0.658–0.712). This comprehensive model demonstrated good sensitivity (70.1%) while maintaining reasonable specificity (58.5%), representing optimal balance for clinical screening applications. The progressive improvement in AUC from 0.586 to 0.685 (Δ = 0.099) represents a clinically meaningful enhancement in predictive accuracy. The final model's performance falls within the “acceptable” to “excellent” discrimination range (AUC > 0.70), making it suitable for clinical risk stratification. The maintained sensitivity above 70% in the comprehensive model ensures adequate case detection for screening purposes, while the reasonable specificity minimizes unnecessary interventions. The substantial superiority of FI over traditional single biomarkers (AUC difference of 0.070–0.077) underscores the value of multidimensional frailty assessment over conventional risk factors alone. The progressive model enhancement suggests that while frailty provides the primary predictive signal, demographic and clinical context significantly augments risk discrimination, supporting the utility of comprehensive geriatric assessment in hearing loss prevention strategies (Fig. 3).

**Fig. 3:**
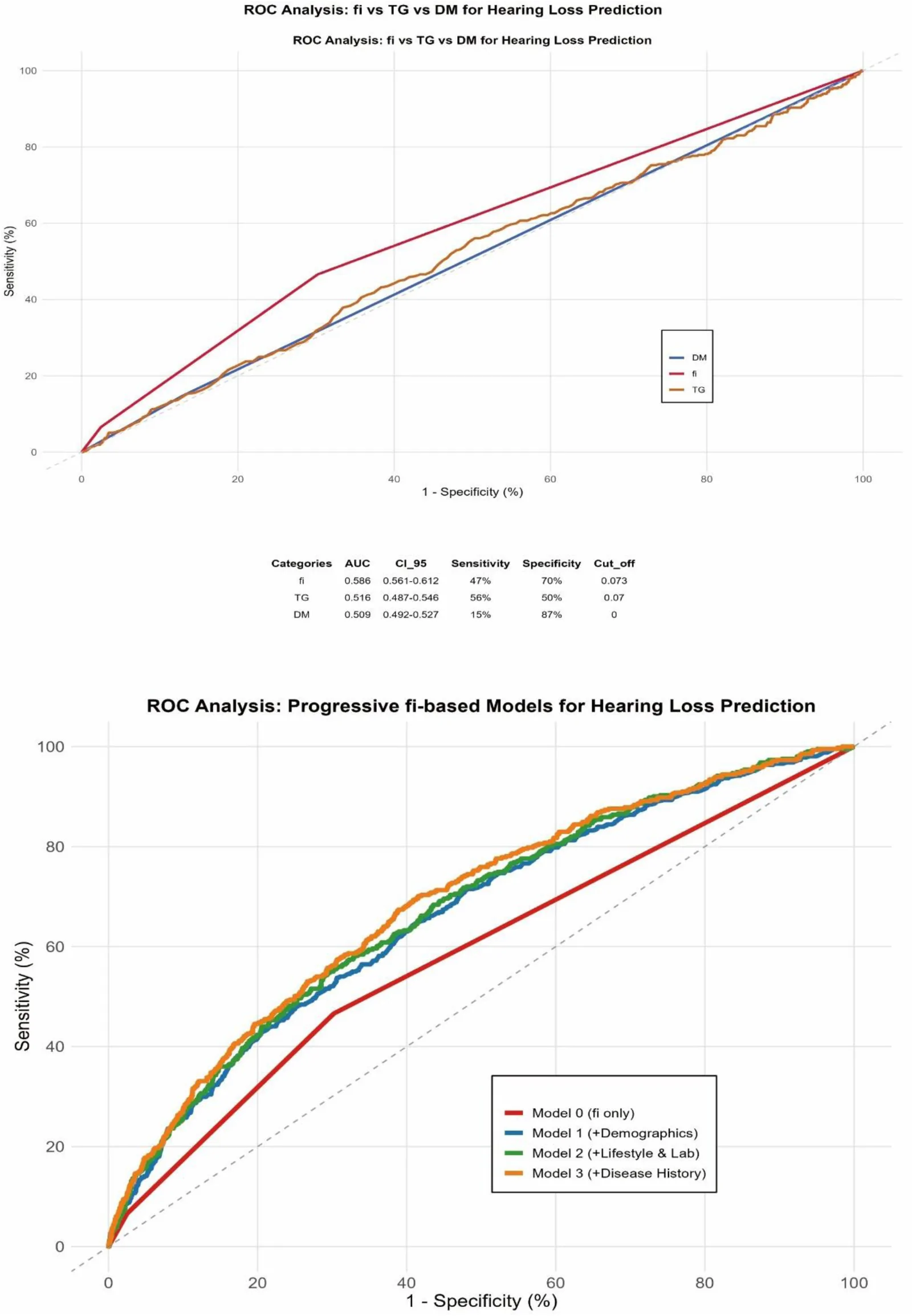
Receiver Operating Characteristic (ROC) curves for predictive models.

### Mendelian randomization analysis

We conducted comprehensive two-sample Mendelian Randomization (MR) analyses to investigate the potential causal relationship between genetically predicted frailty index and various hearing loss phenotypes using summary-level data from large-scale genome-wide association studies. The analyses employed multiple complementary approaches, with Inverse-Variance Weighted (IVW) as the primary method, supplemented by MR-Egger, weighted median, and weighted mode as sensitivity analyses to assess robustness and detect potential horizontal pleiotropy. The IVW analyses provided consistent evidence for a causal effect of higher genetically determined frailty index on increased risk of hearing difficulty across multiple outcome definitions. For hearing difficulty with background noise, the IVW estimate yielded a significant positive association (β = 0.070, SE = 0.021, p = 0.00094) using 15 genetic instruments. Similarly, when examining general hearing difficulty problems from UK Biobank, the effect was substantially larger (β = 0.318, SE = 0.074, p = 1.7 × 10^−5^) with 12 SNPs, indicating a robust causal relationship. However, when age-related hearing impairment was used as the outcome, the IVW estimate showed an unexpected inverse association (β = -0.175, SE = 0.036, p = 1.0 × 10^–6^), suggesting potential phenotypic heterogeneity or distinct etiological pathways for age-specific hearing loss. Sensitivity analyses using the weighted median approach generally corroborated the primary findings, providing additional support for causal inference. For the main hearing difficulty outcomes, weighted median estimates remained positive and statistically significant (β = 0.057, SE = 0.020, p = 0.0055 and β = 0.288, SE = 0.100, p = 0.0041, respectively), demonstrating robustness even when allowing for up to 50% of instruments to be invalid. In contrast, MR-Egger and weighted mode approaches yielded less precise estimates with wider confidence intervals, though the direction of effects generally remained consistent with the primary analyses. Notably, the analyses revealed important heterogeneity across different hearing loss definitions and populations. While most outcomes showed positive causal effects of frailty on hearing impairment, some phenotypes demonstrated inverse or null associations, such as the “hearing difficulty/problems: No” category (IVW β = -0.053, SE = 0.015, p = 0.0003). This heterogeneity likely reflects differences in outcome ascertainment, population characteristics, or underlying biological mechanisms, warranting cautious interpretation of the overall findings (Table S3).

In summary, these MR analyses provide genetic evidence supporting a causal role of frailty in the development of hearing difficulty, with effect sizes varying by specific hearing phenotype. The robustness of findings across multiple MR approaches, combined with minimal evidence of pleiotropy, strengthens the causal inference. However, the observed phenotypic heterogeneity suggests that the relationship between frailty and hearing loss may be complex and context-dependent, highlighting the need for further investigation of underlying mechanisms and potential effect modifiers.

## Discussion

This study presents robust, nationally representative evidence that frailty, measured by the Frailty Index (FI), is an independent, strong, and non-linear predictor of incident hearing loss among middle-aged and older Chinese adults. Drawing on data from the CHARLS cohort and set against global and Chinese trends observed in the GBD 2021 database, our findings reveal a clear and consistent pattern: Individuals with higher levels of frailty are much more likely to experience hearing loss over time, even after adjusting for age, sex, residence, education, comorbidities, metabolic risk factors, and lifestyles. Notably, we observed a substantial dose–response relationship ‒ with risk sharply rising in the first increments of FI, then plateauing at higher frailty scores. Moreover, adding FI to traditional risk models significantly improved the AUC and predictive performance for incident hearing loss. These findings not only extend but deepen current understanding at the intersection of frailty and sensory aging at both epidemiological and mechanistic levels.

Our results substantially advance the literature in several respects. First, while cross-sectional correlations between frailty and sensory (especially hearing) impairments have been previously described, such investigations have often been limited by small sample sizes, single-center recruitment, or insufficient covariate control.^10^^,^^11^ In contrast, our nationally representative cohort with prospective follow-up and rigorous multivariable adjustment establishes temporality and raises confidence in the independent nature of frailty as a risk factor for hearing loss. Furthermore, by leveraging both continuous and categorical analyses of FI and employing advanced regression techniques such as restricted cubic splines, we were able to delineate threshold effects that have direct implications for risk stratification and intervention.

The biological plausibility of the observed association is compelling and likely multifactorial. Frailty and hearing loss are both aging-related syndromes characterized by the progressive accumulation of deficits ‒ reflecting a breakdown of multisystem physiological homeostasis. Key mechanistic pathways linking frailty and hearing impairment likely include increased chronic inflammatory burden (“inflammaging”), microvascular and endothelial dysfunction, oxidative stress, mitochondrial energy depletion, and compromised neuroendocrine regulation.^2^^,^^3^^,^^12^ Vascular risk factors (e.g., hypertension, diabetes, and dyslipidemia) co-aggregate with frailty and are causally implicated in cochlear blood supply compromise, hair cell degeneration, and central auditory pathway impairment.^3^^,^^10^ The FI used in this research also includes neuropsychiatric and cognitive dimensions; accumulating evidence suggests that central presbycusis, reduced cognitive reserve, and higher-order auditory processing deficits are common in frail adults, mediated via neurodegenerative changes in both the auditory cortex and associated brain networks.^13^^,^^14^ Moreover, frail individuals are at greater risk for polypharmacy, including exposure to potentially ototoxic medications, as well as the downstream consequences of sarcopenia and malnutrition ‒ factors with well-described adverse effects on neural and sensory functioning.^15^

Our findings underscore the importance of recognizing frailty and hearing loss as mutually reinforcing phenomena. Previous prospective analyses, such as in the Health ABC Study,^8^ broadly align with our observations, revealing that measured frailty increases the risk of subsequent hearing difficulty. However, our study goes further by demonstrating robust associations across a larger, representative Asian cohort and confirming the improvement in risk prediction when FI is incorporated. Recent cross-sectional studies in China and Japan have also reported similar patterns, but limitations in longitudinal follow-up and the absence of advanced non-linear modeling have precluded the nuanced dose-response characterization presented here.^16^

From a public health and health services perspective, the integration of frailty assessment into hearing health strategies offers distinct advantages. The most immediate is the enhancement of risk stratification and early identification of vulnerable individuals. Hearing loss in older adults is frequently underdiagnosed, particularly in communities with lower health literacy or limited access to specialist assessment^1^. Traditional risk models based mainly on age, comorbidity counts, or clinical suspicion have limited discriminatory ability. Our finding that FI confers meaningful incremental predictive value suggests that routine frailty screening in community and primary care settings could inform more targeted hearing evaluations, optimize resource allocation, and enable timely interventions before irreversible sensory disability develops. Furthermore, our results have substantial implications for prevention and intervention. Recognition of frailty as a systemic, actionable risk state means that broad-based, multidomain interventions ‒ including improved management of vascular, metabolic, psychosocial, and nutritional risk ‒ might simultaneously prevent or delay the onset of both frailty and hearing loss.^17^ Clinical trials of frailty interventions (such as exercise, protein supplementation, or cognitive behavioral programs) have rarely measured auditory outcomes, but our data suggest that such endpoints should be considered in future research. In addition, clinical awareness that hearing loss may be a modifiable component on the frailty trajectory may stimulate earlier use of amplification devices or cognitive/auditory rehabilitation, thereby mitigating not only the primary sensory deficit but also its downstream effects on cognitive decline, mood, and disability.^18^

The significant non-linear relationship and apparent risk threshold observed between FI and hearing loss provides nuanced insight into the window of optimal intervention. Risk rises steeply as frailty progresses from robust to mildly impaired, but further increases in FI above 0.3 confer diminishing additional risk. This pattern suggests that preventive strategies may be most efficacious when targeted at pre-frail or mildly frail populations, rather than focusing interventions on those with advanced frailty in whom reversibility of systemic decline and associated sensory impairment may be limited. This aligns with broader gerontological literature which has shown that much of the impact of frailty interventions ‒ in terms of hospitalizations, falls, disability, and now, as our study suggests, hearing loss ‒ is realized before severe, multi-domain decline has taken hold.^19^

The consistency of our findings across subgroups ‒ including sex, residence, education, chronic disease status, and lifestyle factors ‒ further reinforces the generalizability and relevance of this association.^20^ Although the effect of frailty tended to be more pronounced in rural residents and older age groups, the dose-response pattern was preserved across strata, and no significant statistical interactions emerged. The lack of difference between men and women corroborates the concept that frailty operates as a superseding biological age metric, modulating multiple disease risks regardless of traditional demographic categories.^5^

### Limitations

Despite the strengths of this study, several limitations should be recognized. First, as with all observational and longitudinal cohort studies, residual or unmeasured confounding cannot be fully excluded, even after adjustment for a comprehensive range of covariates. Factors such as subclinical neurological disease, environmental noise exposure, or genetic susceptibility were not directly measured and may have contributed to observed outcomes. Second, incident hearing loss was ascertained via both self-report and partially objective measures, but not all cases were confirmed by pure-tone audiometry, the gold standard in audiometric assessment. This could have resulted in misclassification bias, particularly for mild or early-stage impairments, although sensitivity analyses with varied outcome definitions strengthened the reliability of our results. Third, the construction of the Frailty Index depends on available variables; although we adhered to standard, internationally recommended approaches, limitations in the structure and completeness of the CHARLS dataset may result in FI differences compared to other cohorts. Fourth, the generalizability of our findings to non-Chinese populations, or to groups with distinct environmental, genetic, or healthcare profiles, remains to be further validated through external studies. Fifth, the possibility of selection bias cannot be fully excluded. Participants lost to follow-up or excluded due to missing data may have had a different trajectory regarding frailty and hearing decline, though repeated sensitivity and imputation analyses suggest that this impact was likely minimal. Finally, while the temporal relationship between frailty and subsequent hearing loss is supported by our design, the potential for reverse causality ‒ whereby early, subclinical hearing loss contributes to worsening frailty ‒ cannot be entirely ruled out. Bidirectional relationships deserve further inquiry in future research.

Notwithstanding these limitations, our study possesses several major strengths: a rigorous longitudinal design, comprehensive control for confounding, application of innovative statistical methods, and the fusion of national prospective cohort data with relevant international epidemiological trends. The integration with GBD 2021 findings further contextualizes our results within the broader challenge of population aging and sensory disability at both the global and national levels. Looking ahead, these findings highlight several priority areas for research and practice. Mechanistic investigations are needed to disentangle the cellular, metabolic, and neurocognitive pathways that link frailty and hearing impairment, including possible roles for chronic inflammation, oxidative stress, vascular compromise, and impaired central auditory processing. Randomized controlled trials should examine whether interventions designed to reduce frailty ‒ such as tailored exercise, nutritional support, social engagement, or vascular risk management ‒ can delay or prevent hearing loss, as well as the impact of early hearing intervention on frailty reversal. Policy makers should consider integrating frailty measures into audiological screening algorithms and resource allocation schemes, with a particular focus on high-risk and underserved populations. Finally, international comparative analyses, leveraging standardized frailty measures, are vital to clarifying the universality versus context-specificity of these findings, especially as global populations continue to age at an unprecedented rate.

## Conclusions

In conclusion, this study establishes frailty, as measured by the FI, as a powerful, independent, and non-linear predictor of incident hearing loss in middle-aged and older adults. Our data underscore the value of incorporating frailty assessment into routine preventive, screening, and interventional frameworks for hearing health ‒ not only for individual risk prediction but also as a lever for broader population health improvement. Recognizing the tight biological, clinical, and public health links between frailty and hearing impairment opens new avenues for integrated management and advocacy in the era of global aging.

## ORCID ID

Liang Ren: 0009-0003-9803-7898

Xiangling Jiang: 0000-0002-7121-110X

Maohua Wang: 0000-0002-5985-2430

Xu Zhang: 0009-0009-3257-1012

## Authors’ contributions

Liang Ren and Maohua Wang proposed the concept and design of the study. Xu Zhang and Xiangling Jiang executed the collection and analysis of data. Liang Ren and Xiangling Jiang accomplished the literature search. Liang Ren drafted the main manuscript, while Xu Zhang was responsible for editing and critical revision. Maohua Wang acquired the funding and supervised the study. The final draught of the paper has been approved by all authors once they have read it. The final manuscript was read and approved by all writers.

## Human ethics and consent to participate declarations

All data used in this study are available upon reasonable request from the official CHARLS website (https://charls.pku.edu.cn/index.htm). Professionally trained interviewers and physicians collected the data through standardized questionnaires and physical examinations. The survey was approved by the Ethics Committee of Peking University (IRB00001052-11014). All participants provided written informed consent prior to participation. All methods were conducted in accordance with the relevant guidelines and regulations provided by the official CHARLS protocol. This study followed the STROBE reporting guidelines.

## Funding

No.

## Data availability

The datasets supporting the conclusions of this article are available from multiple public repositories. The Global Burden of Disease Study 2021 (GBD2021) data are publicly available through the Institute for Health Metrics and Evaluation (IHME) Global Health Data Exchange (GHDx) at http://ghdx.healthdata.org/. The China Health and Retirement Longitudinal Study (CHARLS) data are available through the CHARLS official website at http://charls.pku.edu.cn/en after completing the user registration process.

For the Mendelian randomization analyses, Genome-Wide Association Study (GWAS) summary statistics were obtained from publicly available databases. Frailty index GWAS summary statistics were accessed from the UK Biobank through the IEU OpenGWAS Project (https://gwas.mrcieu.ac.uk/). Hearing loss and hearing-related traits GWAS summary statistics were obtained from the UK Biobank and other large-scale biobanks, available through the GWAS Catalog (https://www.ebi.ac.uk/gwas/) and the IEU OpenGWAS database. Additional audiometric and sensory function-related GWAS data were sourced from relevant consortiums including hearing research collaboratives and sensory genomics initiatives.

All datasets are freely accessible to researchers for academic purposes upon agreeing to their respective terms of use and data access agreements. Additional processed datasets, analytical codes, and supplementary materials used in the current study are available from the corresponding author upon reasonable request.

## Declaration of competing interest

The authors declare that they have no known competing financial interests or personal relationships that could have appeared to influence the work reported in this paper.
