# Supplementary material for: Frailty index predicts hearing loss in middle-aged and older adults: A triangulated analysis of global, cohort, and genetic data
Source: Braz J Otorhinolaryngol. 2026 Oct 17;92(6):101919. doi: 10.1016/j.bjorl.2026.101919 (PMC13696800; doi:10.1016/j.bjorl.2026.101919)
Supplement: Supplementary file 1 [file mmc1.docx]

BJORL-D-26-00189_Supplementary Material

**Table S1** Prevalence burden of hearing loss at global and regional levels, 1990‒2021

| **Location** | **Num_1990** | **ASR_1990** | **Num_2021** | **ASR_2021** | **EAPC_CI** |
| --- | --- | --- | --- | --- | --- |
| Andean Latin America | 4001511 (3836720 to 4182975) | 15593.67 (14925.85 to 16337.16) | 9751782 (9331159 to 10213857) | 15556.77 (14918 to 16295.28) | 0.01 (0 to 0.01) |
| Australasia | 3323817 (3216792 to 3440604) | 14567.67 (14090.28 to 15083.77) | 6943434 (6596523 to 7337984) | 14919.31 (14219.44 to 15700.09) | 0.12 (0.08 to 0.16) |
| Caribbean | 4857062 (4649635 to 5093027) | 16888.72 (16182.22 to 17738.71) | 8809247 (8431426 to 9250686) | 16849.19 (16128.51 to 17691.44) | 0 (-0.01 to 0) |
| Central Asia | 9199470 (8808500 to 9623101) | 17241.64 (16526.08 to 18070.3) | 15410561 (14722095 to 16137540) | 17255.91 (16517.53 to 18061.83) | 0 (0 to 0) |
| Central Europe | 24751897 (23678815 to 25937063) | 17361.51 (16625.93 to 18178.91) | 31293101 (29873131 to 32986112) | 17377.53 (16666.89 to 18202.53) | 0 (0 to 0) |
| Central Latin America | 18219277 (17453557 to 19034526) | 16980.45 (16283.11 to 17816.7) | 43546750 (41724482 to 45613784) | 16928.97 (16230.49 to 17726.35) | -0.01 (-0.01 to -0.01) |
| Central Sub-Saharan Africa | 5846193 (5560040 to 6145042) | 17584.13 (16847.17 to 18347.05) | 14993131 (14303040 to 15786492) | 17465.15 (16773.05 to 18230.53) | -0.03 (-0.03 to -0.02) |
| East Asia | 225658993 (214600212 to 238595926) | 22323.49 (21279.58 to 23657.52) | 474146459 (450115386 to 500896774) | 23037.61 (21965.51 to 24286.31) | 0.11 (0.09 to 0.13) |
| Eastern Europe | 46240253 (44228427 to 48461378) | 17516.07 (16789.49 to 18335.19) | 52848284 (50408166 to 55521160) | 17525.17 (16772.93 to 18347.01) | 0 (0 to 0) |
| Eastern Sub-Saharan Africa | 23832152 (22632294 to 25131266) | 21914.26 (20821.64 to 23170.8) | 57995953 (55001959 to 61328812) | 22564.78 (21378.49 to 23936.53) | 0.08 (0 to 0.16) |
| Global | 833403941 (798993045 to 869998858) | 18787.57 (18024.64 to 19629.43) | 1658915808 (1592152094 to 1731065291) | 19532.45 (18770.42 to 20365.05) | 0.11 (0.1 to 0.12) |
| High-income Asia Pacific | 29394462 (28102784 to 30814033) | 14840.23 (14236.87 to 15555.74) | 51868168 (49514907 to 54565900) | 14851.77 (14245.94 to 15526.82) | 0 (0 to 0.01) |
| High-income North America | 49682277 (47052032 to 52425646) | 14924.9 (14162.61 to 15710.11) | 82439282 (78173652 to 87506980) | 14415.62 (13726.21 to 15191.43) | -0.16 (-0.19 to -0.12) |
| High-middle SDI | 191322415 (183316272 to 200584866) | 18491.31 (17728.96 to 19376.64) | 353760541 (338586690 to 371103820) | 19674.12 (18876.49 to 20561.68) | 0.2 (0.2 to 0.21) |
| High SDI | 153833293 (147308391 to 161043243) | 14774.64 (14161.45 to 15441.33) | 258443194 (247044308 to 271060110) | 14947.9 (14338.47 to 15629.02) | 0.03 (0.02 to 0.04) |
| Low-middle SDI | 159933496 (153148069 to 166779339) | 20083.91 (19322.28 to 20918) | 330780393 (317755560 to 344844315) | 19976.99 (19226.34 to 20822.21) | -0.06 (-0.07 to -0.05) |
| Low SDI | 61003607 (58235134 to 63800022) | 19534.63 (18721.67 to 20400.57) | 141266327 (134943882 to 147862540) | 19569.76 (18761.97 to 20456.7) | -0.02 (-0.04 to 0) |
| Middle SDI | 266500314 (254792570 to 278901888) | 20878.51 (19996.35 to 21896.84) | 573385665 (549184081 to 601572289) | 21101.53 (20239.82 to 22065.18) | 0.02 (0.01 to 0.03) |
| North Africa and Middle East | 35301404 (33683939 to 36906176) | 16064.66 (15405.91 to 16774.76) | 84951433 (81295826 to 88766463) | 16006.5 (15343.18 to 16709.67) | -0.01 (-0.02 to -0.01) |
| Oceania | 847331 (808370 to 891179) | 20961.56 (20020.81 to 22014.73) | 2071429 (1981330 to 2178833) | 21118.56 (20181.03 to 22247.43) | -0.04 (-0.06 to -0.02) |
| South Asia | 161615474 (154939328 to 168382922) | 21027.56 (20191.71 to 21884.56) | 353385314 (339882736 to 368297337) | 21059.01 (20304.98 to 21929.06) | -0.05 (-0.07 to -0.03) |
| Southeast Asia | 72422356 (69217268 to 75951328) | 22123.89 (21172.55 to 23201.99) | 157446810 (150212986 to 165425317) | 22263.63 (21290.52 to 23340.41) | -0.03 (-0.05 to -0.02) |
| Southern Latin America | 6620214 (6318310 to 6937293) | 14107.03 (13464.74 to 14783.2) | 11509096 (10993881 to 12109547) | 14078.94 (13468.5 to 14799.13) | -0.01 (-0.01 to 0) |
| Southern Sub-Saharan Africa | 6241762 (5997883 to 6510292) | 17243.04 (16660.7 to 17906.04) | 12209761 (11769340 to 12720579) | 17446.32 (16850.75 to 18096.27) | 0.06 (0.04 to 0.08) |
| Tropical Latin America | 21124023 (20156401 to 22156353) | 18964.33 (18099.07 to 19930.44) | 48109983 (45834947 to 50527612) | 18911.79 (18062.18 to 19844.78) | -0.02 (-0.03 to 0) |
| Western Europe | 64804671 (62160918 to 67815713) | 12743.01 (12236.43 to 13309.55) | 92319568 (88434824 to 96736569) | 12712.04 (12223.76 to 13278.25) | -0.02 (-0.03 to -0.02) |
| Western Sub-Saharan Africa | 19419342 (18382683 to 20417892) | 15877.52 (15188.44 to 16605.48) | 46866263 (44327401 to 49408063) | 15298.5 (14647.96 to 16021.51) | -0.13 (-0.18 to -0.08) |

**Table S2** YLDs burden of hearing loss at global and regional levels, 1990‒2021

| **Location** | **Num_1990** | **ASR_1990** | **Num_2021** | **ASR_2021** | **EAPC_CI** |
| --- | --- | --- | --- | --- | --- |
| Andean Latin America | 108915 (73356 to 154651) | 444.05 (302.62 to 633.46) | 269495 (181365 to 385345) | 439.88 (297.39 to 629.64) | 0.03 (-0.01 to 0.06) |
| Australasia | 97561 (65605 to 137525) | 425.84 (287.34 to 601.16) | 210141 (140635 to 301646) | 426.79 (283.76 to 615.54) | 0.13 (0.06 to 0.2) |
| Caribbean | 139487 (94123 to 197890) | 496.63 (335.24 to 704.63) | 257853 (175103 to 366548) | 491.33 (332.82 to 698.2) | -0.02 (-0.03 to -0.02) |
| Central Asia | 270093 (181953 to 383388) | 521.12 (354.67 to 738.56) | 433272 (290543 to 615474) | 516.51 (349.01 to 733.22) | -0.02 (-0.03 to -0.01) |
| Central Europe | 738906 (501497 to 1056938) | 526.2 (356 to 749.3) | 988908 (673283 to 1404781) | 521.24 (351.91 to 740.53) | -0.01 (-0.02 to -0.01) |
| Central Latin America | 505052 (339743 to 715356) | 496.21 (337.08 to 706.6) | 1233118 (832386 to 1765425) | 490.85 (331.64 to 702.38) | -0.02 (-0.03 to -0.02) |
| Central Sub-Saharan Africa | 201500 (136715 to 277701) | 631.69 (437.16 to 885.83) | 500922 (342450 to 700936) | 618.01 (429.86 to 868.16) | -0.08 (-0.09 to -0.08) |
| East Asia | 6159814 (4109515 to 8833968) | 644.41 (432.03 to 917.52) | 13875897 (9424876 to 19740103) | 689.13 (466.24 to 975.49) | 0.27 (0.23 to 0.31) |
| Eastern Europe | 1420503 (968434 to 2023935) | 546.2 (372.68 to 768.26) | 1667522 (1133732 to 2369896) | 539.65 (366.73 to 763.39) | -0.03 (-0.03 to -0.03) |
| Eastern Sub-Saharan Africa | 815006 (559877 to 1139643) | 782.06 (544.2 to 1102.34) | 1920372 (1319008 to 2684493) | 792.47 (551.31 to 1119.63) | 0.02 (-0.07 to 0.11) |
| Global | 25168705 (17009530 to 35624098) | 581.14 (394.41 to 817.08) | 50909379 (34479898 to 71817592) | 605.99 (410.64 to 849.99) | 0.13 (0.12 to 0.14) |
| High-income Asia Pacific | 773298 (512994 to 1126066) | 399.17 (265.78 to 577.17) | 1575402 (1066883 to 2232192) | 394.13 (262.74 to 570.82) | 0 (-0.01 to 0.02) |
| High-income North America | 1693372 (1150957 to 2410472) | 497.14 (338.97 to 708.3) | 2826053 (1926704 to 4017610) | 475.39 (322.81 to 675.71) | -0.19 (-0.24 to -0.13) |
| High-middle SDI | 5540507 (3736797 to 7879621) | 552.21 (374.72 to 778.56) | 10633304 (7215926 to 15101985) | 591.51 (399.74 to 833.47) | 0.25 (0.23 to 0.27) |
| High SDI | 4581721 (3106702 to 6517512) | 433.29 (293.66 to 617.32) | 8068098 (5474972 to 11412651) | 436.91 (294.89 to 623.08) | 0.05 (0.04 to 0.07) |
| Low-middle SDI | 5118107 (3521196 to 7124198) | 667.27 (462.45 to 929.99) | 10315177 (7058044 to 14493267) | 648.07 (446 to 904.89) | -0.14 (-0.16 to -0.13) |
| Low SDI | 2058931 (1418140 to 2863799) | 683.63 (475.63 to 962.03) | 4645780 (3194267 to 6476211) | 670.59 (465.3 to 942.43) | -0.1 (-0.12 to -0.07) |
| Middle SDI | 7845275 (5296250 to 11123580) | 641.34 (434.78 to 902.08) | 17208025 (11621731 to 24525788) | 655.79 (443.3 to 923.52) | 0.08 (0.06 to 0.09) |
| North Africa and Middle East | 1223280 (837564 to 1720822) | 583.39 (404.27 to 824.01) | 2769020 (1887761 to 3893896) | 556.15 (381.77 to 786.16) | -0.15 (-0.15 to -0.15) |
| Oceania | 22627 (15076 to 32369) | 595.81 (399.89 to 850.3) | 54117 (35907 to 78223) | 595.04 (397.17 to 855.23) | -0.05 (-0.06 to -0.03) |
| South Asia | 5135963 (3551102 to 7119839) | 700.57 (485.73 to 974.34) | 10948274 (7509433 to 15306119) | 679.94 (468.48 to 948.19) | -0.16 (-0.18 to -0.14) |
| Southeast Asia | 2300357 (1565595 to 3220456) | 714.31 (485 to 1000.2) | 4744898 (3191593 to 6720147) | 705.11 (478.5 to 988.88) | -0.09 (-0.11 to -0.08) |
| Southern Latin America | 195464 (132240 to 278120) | 424.23 (288.46 to 601.57) | 349265 (238754 to 497609) | 417.25 (284.07 to 593.83) | -0.04 (-0.05 to -0.03) |
| Southern Sub-Saharan Africa | 172644 (117086 to 245604) | 499.51 (339.82 to 710.85) | 326445 (220558 to 465354) | 497.8 (339.39 to 708.99) | 0.03 (0.02 to 0.04) |
| Tropical Latin America | 609377 (407273 to 873856) | 575.43 (388.25 to 825.7) | 1425237 (955035 to 2053117) | 569.45 (383.77 to 820.36) | -0.05 (-0.1 to 0) |
| Western Europe | 1861544 (1244663 to 2681478) | 349.15 (235.04 to 503.44) | 2827673 (1911153 to 3998103) | 345.79 (231.88 to 499.27) | 0.04 (0.02 to 0.06) |
| Western Sub-Saharan Africa | 723944 (495800 to 1002065) | 597.81 (415.61 to 838.07) | 1705496 (1161320 to 2367441) | 567.75 (394.96 to 795.27) | -0.19 (-0.24 to -0.13) |

**Table S3** Causal effects of frailty index on hearing impairment and hearing difficulties: mendelian randomization results.

| **Exposure_ID** | **Outcome_ID** | **Outcome** | **Exposure** | **Method** | **Nsnp** | **β** | **Se** | **p-val** |
| --- | --- | --- | --- | --- | --- | --- | --- | --- |
| ebi-a-GCST90020053 | ebi-a-GCST90012115 | Age-related hearing impairment | Frailty index | Inverse variance weighted | 13 | -0.17501 | 0.04 | 1.39E-06 |
| ebi-a-GCST90020053 | ebi-a-GCST90013911 | Hearing difficulty problems | Frailty index | Inverse variance weighted | 12 | 0.32 | 0.07 | 1.66E-05 |
| ebi-a-GCST90020053 | ebi-a-GCST90013961 | Hearing difficulty problems | Frailty index | Inverse variance weighted | 12 | 0.32 | 0.07 | 1.67E-05 |
| ebi-a-GCST90020053 | ukb-a-257 | Hearing difficulty/problems: Yes | Frailty index | Inverse variance weighted | 12 | 0.06 | 0.02 | 2.00E-04 |
| ebi-a-GCST90020053 | ukb-b-18275 | Hearing difficulty/problems with background noise | Frailty index | Inverse variance weighted | 15 | 0.07 | 0.02 | 9.00E-04 |
| ebi-a-GCST90020053 | ukb-r2-2247_0 | Hearing difficulty/problems: No | Frailty index | Inverse variance weighted | 11 | -0.05 | 0.02 | 2.00E-04 |
| ebi-a-GCST90020053 | ukb-r2-2247_1 | Hearing difficulty/problems: Yes | Frailty index | Inverse variance weighted | 11 | 0.05 | 0.02 | 2.00E-04 |
| ebi-a-GCST90020053 | ukb-r2-2257 | Hearing difficulty/problems with background noise | Frailty index | Inverse variance weighted | 14 | 0.09 | 0.02 | 5.25E-05 |

**Figure S1** Subnational distribution of hearing loss and frailty in China (GBD 2021).

**Figure S2** Nonlinear association between frailty index and hearing loss risk.

**Figure S3** Subgroup analyses of the association between frailty index and hearing loss.
